# A case of vasculitis, retinitis and macular neurosensory detachment presenting post typhoid fever

**DOI:** 10.1186/s12348-014-0023-y

**Published:** 2014-09-18

**Authors:** Nidhi Relhan, Avinash Pathengay, Thomas Albini, Krishna Priya, Subhadra Jalali, Harry W Flynn

**Affiliations:** 1 Department of Ophthalmology, Bascom Palmer Eye Institute, University of Miami Miller School of Medicine, Miami 33136, FL, USA; 2 Srimati Kannuri Santhamma Centre for Vitreo-Retinal Diseases, L V Prasad Eye Institute, Kallam Anji Reddy Campus, Hyderabad 500034, Telangana, India; 3 Retina and Uveitis Department, L V Prasad Eye Institute, GMR Varalaxmi Campus, Visakhapatnam 530 040, Andhra Pradesh, India

**Keywords:** Post typhoid retinitis, Neuroretinitis, Immune-mediated retinitis, Post fever retinitis

## Abstract

**Background:**

Ocular and extraocular immune-mediated phenomena are known to occur following febrile illness. Vasculitis, retinitis and neurosensory detachment are not well-recognized sequelae of typhoid fever.

**Findings:**

We report a case of vasculitis, retinitis and macular neurosensory detachment presenting post typhoid fever. A 27-year-old female presented with decreased vision in right eye with history of typhoid fever (treated adequately 6 weeks prior). Her best corrected visual acuity in right eye was 20/125, N36. Fundus showed a patch of vasculitis and retinitis superior to the disc associated with macular neurosensory detachment and disc pallor. With oral steroids, the inflammation resolved and visual acuity improved to 20/20 at 6 weeks.

**Conclusions:**

Immune-mediated vasculitis and retinitis following typhoid fever may respond well to systemic steroids.

## Findings

### Introduction

Typhoid fever is caused by *Salmonella typhi*. It leads to enteric fever, septicemia and gastroenteritis. *Salmonella* can rarely affect the eye either by direct infection or rarely by immune-mediated mechanism. Hersing and Duke-Elders [1] reported typhoid-related uveal complications including iritis, retinal hemorrhage, choroiditis, endophthalmitis and panophthalmitis. Our group published [2],[3] late-onset endogenous endophthalmitis post typhoid fever resolution. In this manuscript we report a patient who presented with retinitis and had a history of typhoid fever, beginning 6 weeks prior to presentation.

## Case report

**Figure 1:**
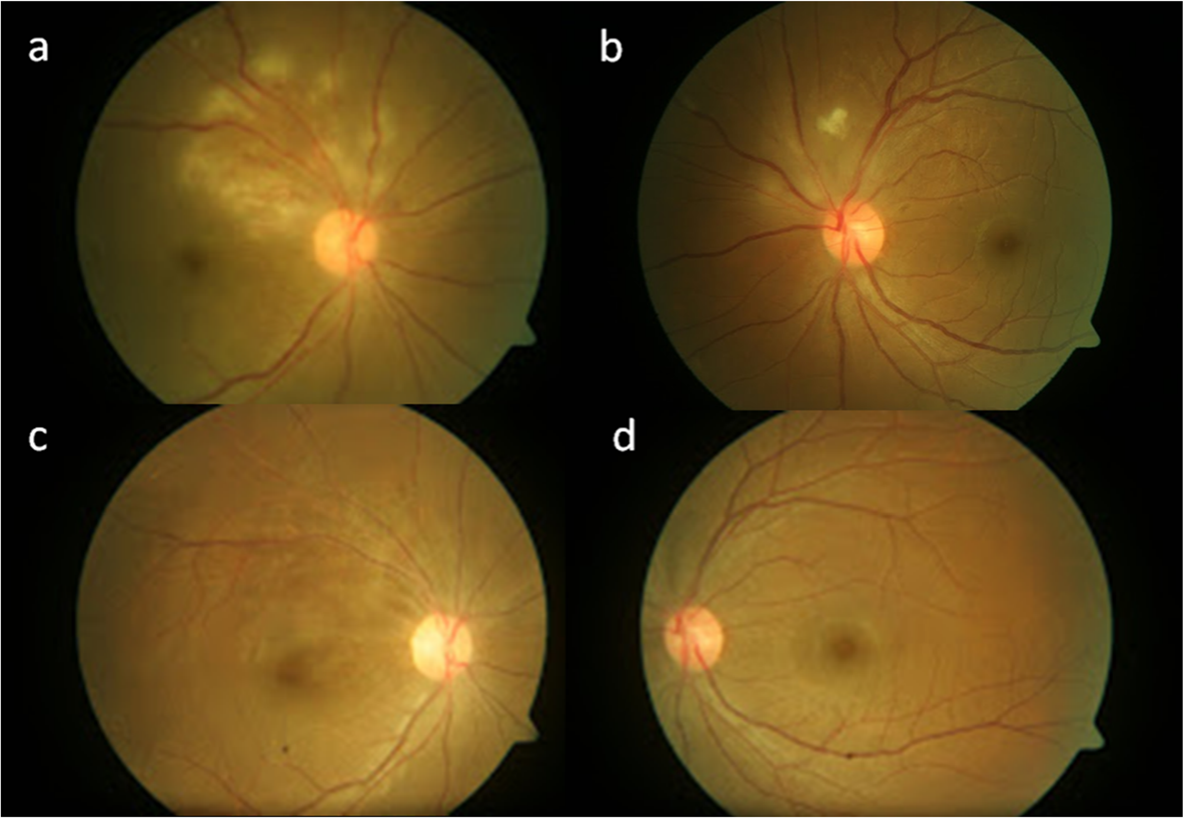
**Fundus photographs of the right and left eyes.** At presentation (**a, b** respectively) showing vasculitis and retinitis patch (right > left). At 6 months follow-up, the fundus photographs of the right and left eyes (**c, d** respectively) show complete resolution of macular edema with residual RPE changes.

**Figure 2:**
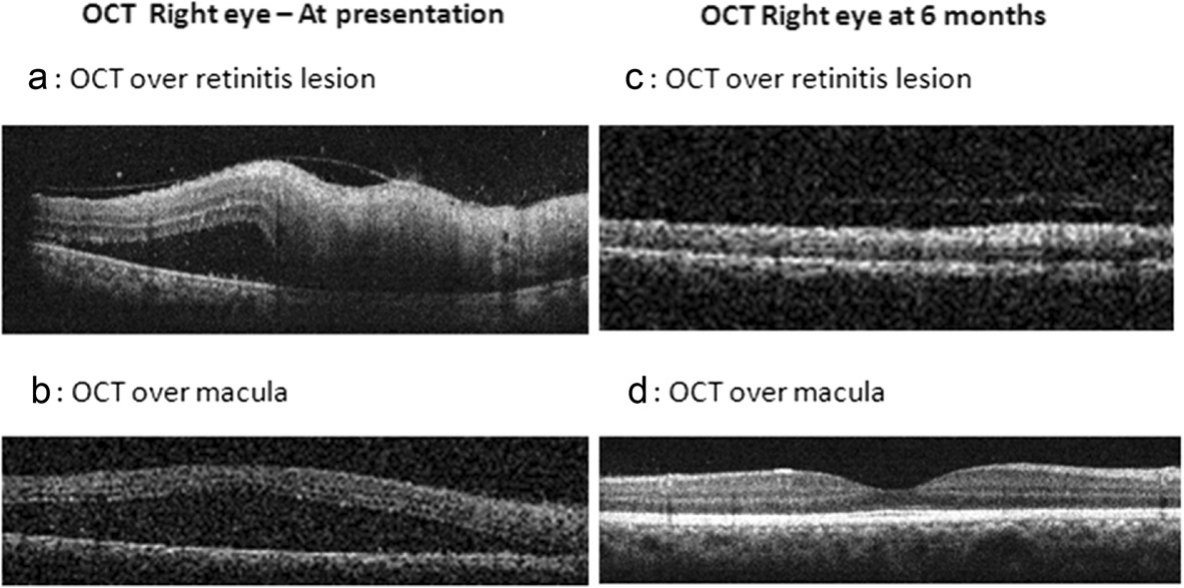
**Optical coherence tomography (Optovue) of the right eye.** At presentation showing inner retinal layer hyperreflectivity with backscattering over the lesion **(a)** and presence of large subfoveal neurosensory detachment **(b)** in the macula. At 6 months follow-up, showing residual thinning of inner retinal layers over the lesion **(c)** and complete resolution of subfoveal neurosensory detachment **(d)** in the macula.

A 26-year-old Indian female presented in urban southern India, at LV Prasad Eye Institute, Hyderabad, with sudden, painless decreased vision in the right eye for 20 days associated with floaters. She gave a past history of typhoid fever 6 weeks prior to presentation. Treatment and diagnostic details of the past typhoid fever were as follows: positive Widal test with significant titers for ‘O’ antigen (1:320) and ‘H’ antigen (1:40) while ‘AH’ and ‘BH’ antigens were non-reactive. Two weeks following the onset of fever, she initiated treatment with oral ofloxacin 400 mg twice daily for 14 days; following which, the fever resolved. She began to experience decreased vision 4 weeks after the onset of treatment. On ophthalmic examination, her best corrected visual acuity was 20/125, N36 in the right eye and 20/20, N6 in the left eye. Anterior segment examination including slit lamp biomicroscopy was unremarkable except for the presence of grade 1 RAPD in the right eye. Right eye colour vision was totally defective - no colour plates Vs 17/19 in the left eye (using Ishihara's pseudoisochromatic chart). Fundus examination in the right eye (Figure 1a) revealed clear media with slight disc pallor with area of vasculitis superior to disc associated with multiple whitish fluffy areas of deep retinitis (4 to 5 disc diameter) and a large neurosensory detachment in the macular area. This was seen as a highly reflective and disorganized inner retinal layer with back scattering and underlying serous retinal detachment on optical coherence tomography (OCT) (Figure 2a,b). The left eye (Figure 1b) had clear media, normal disc and foveal reflex, one discrete cotton-wool spot superior to the disc and a nasal area of retinal venous sheathing. The patient was offered diagnostic anterior chamber paracentesis but she declined. Baseline workup was done which was found to be negative for HIV, tuberculosis, syphilis, connective tissue disorders, SLE and rheumatoid arthritis. Diagnoses of bilateral post typhoid fever retinitis (possibly immune-mediated) and right eye macular neurosensory detachment were made. In consultation with the patient and an internist, she started prednisolone (1 mg/kg body weight/day). Steroids were tapered over 2 months with regular monitoring. At 6 weeks, she had recovered her visual acuity to 20/20, N6 in both eyes. At 6 months follow-up (Figure 1c,d) the fundus examination in both eyes showed complete resolution of all retinal lesions with pigmentary changes and additional mild disc pallor in the right eye. OCT at 6 months showed residual thinning of inner retinal layers over the lesion (Figure 2c) along with complete resolution of subfoveal neurosensory detachment (Figure 2d) in the macula.

### Discussion and review of literature

This case demonstrates resolution of immune-mediated retinitis following typhoid fever. A viral etiology cannot be completely ruled out. The lesions in this case were not peripheral, did not exhibit circumferential spread, did not involve the arterioles and were not associated with prominent anterior or posterior cellular reaction. Given the timing, 6 weeks following the onset of typhoid fever and significant improvement without antiviral treatment, post typhoid immune-mediated retinitis seems the most likely diagnosis.

Literature review reveals minimal data related to typhoid fever causing this type of pathology [4]-[6]. Anecdotal similarly reported [5] cases have been assumed to be due to retinal infiltration of immune origin. Pathogenesis of immune-mediated vasculitis could be attributed to post infectious immunologic effects which may lead to an immune response that reacts to self-antigens (for example, heat shock protein and myelin basic protein) or homology between retinal antigens and microbial peptides (similarity between S antigen and microbial peptides like yeasts, *Escherichia coli*, and hepatitis B virus) or molecular mimicry leading to autoimmunity (S antigen and interphotoreceptor retinoid binding protein - IRBP) [7]. Diagnosis of immune-mediated retinitis is often clinical, based on past history of a febrile illness (4 to 6 weeks prior) and is supplemented by laboratory workup. Retinitis-occurring post febrile illnesses have been reported after malaria, viral fevers, Chickungunya fever and also in non-infectious immune disorders (Behcet's disease, intraocular lymphoma) [5],[8]. Management of such pathology remains controversial due to lack of published literature. Spontaneous resolution is possible. Mild cases resolve without treatment, while severe cases may need a course of corticosteroids. In our case, steroids were prescribed in view of inflammation involving the disc and macula leading to profound decrease in vision. In conclusion, though rare, one can encounter cases of non-infectious, immune-mediated retinitis after resolution of typhoid febrile illnesses that may necessitate the use of steroids in severe cases.

## Endnote

This paper was presented at Hyde Park Session in Andhra Pradesh Ophthalmology Conference (APOC) December 2012 at Guntur, India.

## Competing interests

The authors declare that they have no competing interests (financial or non-financial).

## Authors' contributions

This case was managed by NR while working at L V Prasad Eye Institute, Hyderabad, India. NR wrote the manuscript under the guidance of SJ and AP. KP was involved in the acquisition of data and drafting of the manuscript. TA and HWF helped to look for the relevant literature, understand the concept better and did the critical revision of manuscript. All the authors have read and given final approval for the manuscript submitted.

## Authors' information

NR is doing a research fellowship at Bascom Palmer Eye Institute, Miami FL, USA. She has worked at L V Prasad Eye Institute, Hyderabad, India. AP and SJ are consultants at the Department of Vitreo-Retina, L V Prasad Eye Institute, India. TA and HWF are consultants at the Department of Ophthalmology, Bascom Palmer Eye Institute, University of Miami Miller School of Medicine, Miami, FL, USA.
